# Regulation of cell-cell fusion by nanotopography

**DOI:** 10.1038/srep33277

**Published:** 2016-09-12

**Authors:** Jagannath Padmanabhan, Michael J. Augelli, Bettina Cheung, Emily R. Kinser, Barnett Cleary, Priyanka Kumar, Renhao Wang, Andrew J. Sawyer, Rui Li, Udo D. Schwarz, Jan Schroers, Themis R. Kyriakides

**Affiliations:** 1 Center for Research on Interface Structures and Phenomena, Yale University, New Haven, CT 06520, USA; 2 Dept. of Biomedical Engineering, Yale University, New Haven, CT 06520, USA; 3 Dept. of Mechanical Engineering and Materials Science, Yale University, New Haven, CT 06520, USA; 4 IBM Semiconductor Research and Development Center/IBM Corporate Patent Engineer, IBM, Hopewell Junction, New York 12533, United States; 5 Department of Pathology, Yale University, New Haven, CT 06520, USA

## Abstract

Cell-cell fusion is fundamental to a multitude of biological processes ranging from cell differentiation and embryogenesis to cancer metastasis and biomaterial-tissue interactions. Fusogenic cells are exposed to biochemical and biophysical factors, which could potentially alter cell behavior. While biochemical inducers of fusion such as cytokines and kinases have been identified, little is known about the biophysical regulation of cell-cell fusion. Here, we designed experiments to examine cell-cell fusion using bulk metallic glass (BMG) nanorod arrays with varying biophysical cues, i.e. nanotopography and stiffness. Through independent variation of stiffness and topography, we found that nanotopography constitutes the primary biophysical cue that can override biochemical signals to attenuate fusion. Specifically, nanotopography restricts cytoskeletal remodeling-associated signaling, which leads to reduced fusion. This finding expands our fundamental understanding of the nanoscale biophysical regulation of cell fusion and can be exploited in biomaterials design to induce desirable biomaterial-tissue interactions.

Fusion of cells to form multinucleated giant cells constitutes an integral component of various physiological processes including the biomaterial-induced foreign body response (FBR)^1^,^2^,^3^,^4^. For example, fusion of macrophages on the surface of biomaterials results in the formation of foreign body giant cells (FBGCs), which contribute to the progression of biomaterial-induced FBR^4^,^5^,^6^,^7^,^8^,^9^. Similarly, fusion of myoblasts leads to the formation of multinucleated myotubes in a process called myogenesis^10^,^11^. Myogenesis contributes to muscle development during embryogenesis as well as muscle regeneration during tissue repair. Modulation of cell fusion by biochemical signals such as cytokines, kinases and GTPases has been studied extensively^7^,^9^,^11^,^12^,^13^,^14^. It has also been reported that specific cell co-cultures can lead to cell fusion. For example, co-culture of macrophages and fibroblasts can lead to formation of fibroblast-derived multinucleated giant cells^15^. Additionally, soluble polymers such as polyethylene glycol (PEG) have also been shown to induce cell-cell fusion *in vitro*^16^,^17^.

Unlike biochemical induction, the biophysical regulation of cell fusion is not well understood. Biophysical cues such as topography and stiffness are ubiquitous in the native ECM and implanted biomaterials^5^,^18^. Biophysical regulation of cell behavior has been extensively studied using micro/nanostructured materials generated by employing lithography-based techniques as well as alternate methods such as modified polymer demixing/spin-coating and colloidal chemistry based techniques^19^,^20^,^21^. Such patterned materials have been employed to investigate the effect of topography and stiffness on multiple cell types including fibroblasts, smooth muscle cells and stem cells^5^,^22^,^23^,^24^,^25^,^26^,^27^. Exploring the effect of these biophysical parameters on cell fusion could uncover unknown aspects of cell physiology, already present in nature. In this regard, micro- and nanopatterned biomaterials have been employed to investigate the effect of topography on cell fusion^5^,^24^,^25^,^28^,^29^,^30^. Specifically, micron-sized ridges or gratings have been shown to increase cell alignment and cell fusion in myoblasts and macrophages^29^,^30^,^31^. Additionally, it has been suggested that micro islands and nanotubes can be used to modulate cell fusion^26^,^32^,^33^. While these studies provide evidence that topography can induce changes in cell fusion, there is no consensus on how the fusogenic capacity of primary cells changes in response to the biomechanical cues presented by nanopatterned biomaterials. Specifically, our current understanding of cell fusion cannot distinguish between topographical features and stiffness due to experimental limitations.

Additionally, most of the studies described above employed polymers to investigate the effect of topography on cell fusion. Polymers constitute a versatile, easily processable class of materials and have been instrumental to our understanding of cell-biomaterial interactions. Metals and metallic alloys constitute another class of materials often used to develop biomaterials for bone replacements, cardiovascular stents and other applications. Metals display high elastic moduli and corrosion resistance, and have been traditionally used for structural implants^34^. However, the practical applicability of nanopatterning strategies with metals is restricted due to intrinsic limits in processability of high-strength metals. Hence, there is a clinical need for biocompatible, high strength materials that can be processed at various length scales including the nanoscale. Bulk metallic glasses (BMGs), metallic alloys with an amorphous atomic structure, constitute a class of materials ideally suited to address the clinical need described above^35^,^36^,^37^,^38^,^39^. BMGs possess metal-like properties such as high strength and elastic moduli, and increased resistance to corrosion and wear^35^. Unlike conventional metals, BMGs lack a microstructure comprised of crystals, decorated with dislocations and separated by grain boundaries. Instead, their atomic structure results in a homogenous and isotropic material with unique properties. For example, BMGs exhibit a unique temperature-dependent mechanical behavior, which enables polymer-like processability at temperatures higher than the critical BMG-specific glass transition temperature^40^,^41^. Therefore BMG alloys combine metal-like strength and stiffness with polymer-like processability. Nanopatterning of biomaterials to engineer cellular response has been suggested for polymer-based materials previously^5^. Development of BMG nanorods enable the application of nanopatterning-based strategies with high strength and stiffness materials. We recently demonstrated that thermoplastic forming of Platinum-based BMGs (Pt_57.5_Cu_14.7_Ni_5.3_P_22.5_, Pt-BMGs) above its glass transition temperature can be exploited to generate a variety of precise nanorod arrays with feature sizes below 100 nm^37^.

In the present study, we employed thermoplastic forming to generate Pt-BMG nanorod arrays to investigate the effect of topographical features and stiffness on cell fusion. Thermoplastic forming of BMGs is uniquely suited to generate surfaces to study these properties. The forming process involves heating the BMG to its supercooled liquid region, the temperature range where the glass relaxes into a liquid before it eventually crystallizes. Pressure is applied to mold the BMG into commercially available nanoporous alumina molds. The porous morphology of the alumina mold determines the topographical parameters such as nanorod diameter and inter-nanorod spacing (pitch).

Analysis of fusion revealed that various nanopattern arrays could reduce fusion, with 55 nm nanorods displaying maximum attenuation. Nanorod length was independently manipulated to generate identical nanorod arrays in terms of diameter and pitch with distinct aspect ratios between 7 and 2, which corresponded to a forty-fold difference in nanorod stiffness. Despite this variation in individual nanorod stiffness, these nanorod arrays induced a similar reduction in cell fusion, indicating that topographical cues can override biochemical signals to attenuate fusion. Nanotopography-induced attenuation of cell fusion was mediated by compromised activation of p38 MAP kinase and inhibition of cytoskeletal remodeling.

## Results

### Biophysical regulation of cell-cell fusion: Topography vs. Stiffness

**Figure 1:**
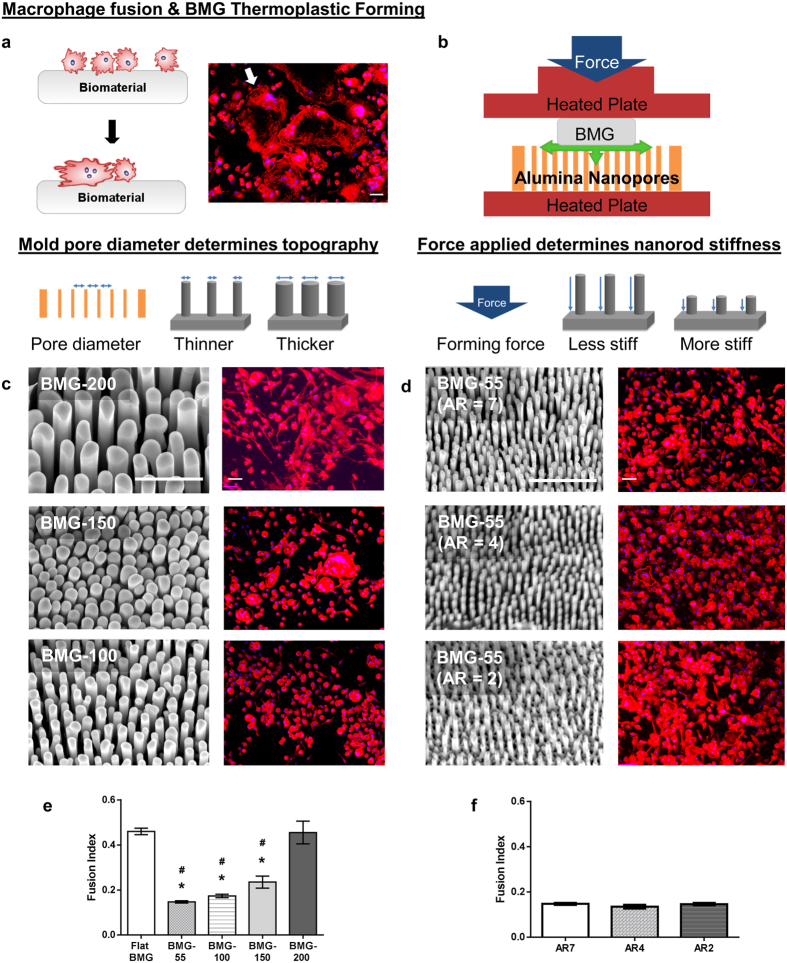
Macrophage fusion on BMG nanorod arrays: Isolating the role of topography vs. stiffness in modulating macrophage behavior. (**a**) (Left) Schematic of the macrophage fusion. Macrophages can fuse to form FBGCs on the biomaterial surface. (Right) IL-4 treated macrophages cultured on flat BMGs for 10 days fuse to form FBGCs. Cells stained for F-actin (phalloidin) and nuclei (DAPI). White arrow indicates a single FBGC in the image. Scale bar = 70 μm. (**b**) Schematic of formation of nanorods by thermoplastic forming of BMGs using nanoporous alumina molds. Porosity of alumina molds determines nanorod diameter while force applied determines nanorod height. (**c**) (Left) Representative SEM images of nanopatterned BMGs. (BMG-55s, BMG-100s, BMG-150s and BMG-200s refer to nanopatterned BMGs with nominal nanorod diameter of 55, 100, 150 & 200 nm, Supplementary Fig. S1) Scale bar = 1 μm. (Right) IL-4 treated macrophages cultured on BMGs for 10 days, stained for F-actin (phalloidin) and nuclei (DAPI). Scale bar = 70 μm. (**d**) (Left) Representative SEM images of BMG-55s with varying nanorod aspect ratios and therefore different stiffness. Scale bar = 1 μm. (Right) IL-4 treated macrophages on BMGs cultured for 10 days, stained for F-actin (phalloidin) and nuclei (DAPI). Scale bar = 70 μm. (**e,f**) Fusion index (FI) of macrophages on flat and nanopatterned BMGs. FI was defined as the ratio of number of fused nuclei to total nuclei and was quantified in ImageJ. *represents significant differences as compared to flat BMGs. ^#^represents significant differences as compared to BMG-200s. Error bars represent standard error mean (SEM). (ANOVA with Tukey’s posthoc analysis, (n ≥ 3). p ≤ 0.05 for significance.) Nanorods 55 nm in diameter significantly decreased fusion while changes in aspect ratio did not.

In the present study, cell-cell fusion of primary bone-marrow derived macrophages cultured on Pt-BMG nanorod arrays (Supplementary Fig. S1) was biochemically induced using IL-4. Macrophages on flat control BMGs displayed extensive fusion, forming large multinucleated FBGCs that accounted for 42% of all the nuclei (Fig. 1a). Cell fusion on BMG nanorod arrays was compromised to various extents depending on surface topography (Fig. 1c–e). Moreover, analysis of changes in cell adhesion density induced by nanorod arrays revealed that the decrease in cell-cell fusion observed was not correlated to reduced cell adhesion. For example, the average number of total nuclei on 55 nm nanorod arrays and 100 nm nanorod arrays was similar to that on Flat BMGs (Supplementary Fig. S1). Despite similar adhesion density, cell-cell fusion was reduced on these nanorod arrays. All nanorod arrays tested displayed a significant reduction in FBGC size as well as average number of nuclei per FBGC (Supplementary Fig. S1). 55 nm nanorod arrays, termed BMG-55s, were the most effective in attenuating cell-cell fusion and were chosen for further experimentation (Fig. 1c–e, Supplementary Fig. S1). To assess the effect of nanorod stiffness in isolation, BMG-55s with varying nanorod lengths were fabricated. Forming pressure was altered to increase the filling depth, which increases aspect ratio and hence generates nanorod arrays with similar topography (diameters and pitch), but varied aspect ratios and stiffness. A nanorod aspect ratio of seven for BMG-55s was used, which results in a stiffness of 3 nN/nm. In addition, BMG-55s with an average nanorod aspect ratio of 4 and 2 were generated with nanorod stiffness of 14 nN/nm and 114 nN/nm respectively (Supplementary Fig. S2). The average nanorod diameter for BMG-55s with different aspect ratios was 64 ± 10 nm. Hence, BMG-55s with varying aspect ratios represented nanorod arrays with identical diameters and pitch (topographical features) but a 40-fold difference in nanorod stiffness. Primary macrophages were seeded on BMG-55s and cell fusion was analyzed, which revealed that change in nanorod stiffness does not significantly alter macrophage fusion (Fig 1d,f). Taken together, these results imply that nanorod arrays with similar topographical cues (nanorod diameter and pitch) can override biochemical signals, independent of changes in nanorod stiffness cues, to regulate cell fusion (Fig. 1c–f).

Our results reveal that some macrophage responses to nanotopography occur independent of substrate stiffness. It is also reported in the literature that cell fusion is unique in that it responds to changes in topography, but not stiffness^18^,^42^. Other cell types respond to changes in substrate stiffness by altering their morphologies and functions^42^,^43^. For example, fibroblasts respond to decreasing substrate stiffness by assuming a more rounded morphology and reducing collagen production^37^,^43^. An explanation for the differences between macrophages and other cell types may be found in the distinct nature of cell-substrate adhesions formed by these cells. Fibroblasts form stable focal adhesions, which anchor the cells to the underlying substrate^37^,^44^. In contrast, stable focal adhesions are missing in macrophages, which use short adhesions and podosomes to interact with the substrate^45^,^46^. Furthermore, it is important to note that in addition to topography and stiffness, nanomaterial shape constitutes a consequential biomechanical property. Specifically, it has been previously reported that ridges or trenches increase cell fusion^29^,^30^. Our results show that nanorod arrays can attenuate macrophage fusion. In this regard, their response is similar to that observed in other inflammatory cells interacting with nanofibers and nanoparticles and is dependent on nanomaterial shape^5^,^47^,^48^,^49^,^50^,^51^.

### Nanotopography-induced attenuation of cytoskeletal remodeling results in reduced fusion

**Figure 2:**
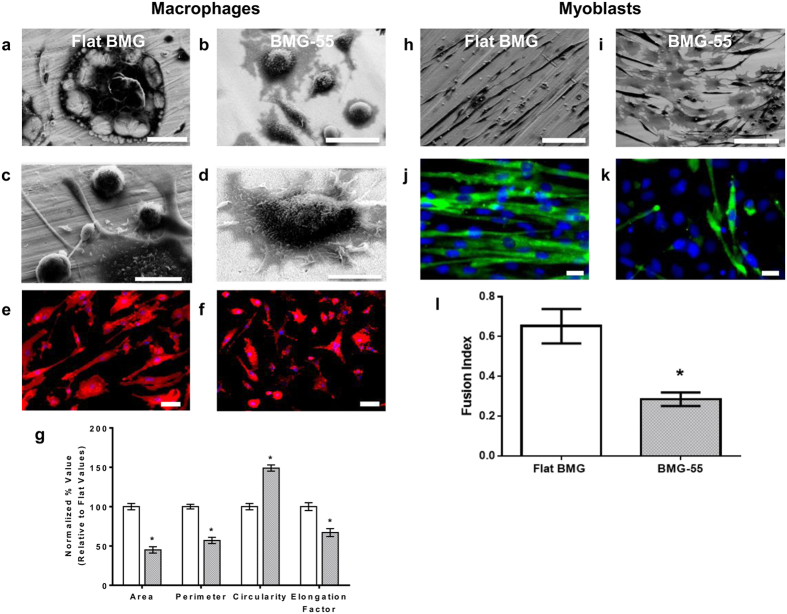
Nanotopography-induced restriction in cytoskeletal remodeling results in decreased cell fusion. (**a–d**) Representative SEM images of IL-4 treated macrophages on flat BMG and BMG-55 cultured for 10 days. Scale bar for a = 100 μm, b = 50 μm. c, d = 20 μm. (**e,f**) Representative images of IL-4 treated macrophages on flat BMGs and BMG-55s at Day 2 timepoint, stained for F-actin (phalloidin) and nuclei (DAPI). Scale bar = 21 μm. (**g**) Quantification of parameters of macrophage morphology on flat (white) and BMG-55 (gray) samples. Cells on flat BMGs display an elongated, well-spread morphology, while cells on BMG-55s show significantly lower cell area and elongation. (**h,i**) Representative SEM images of transferrin/insulin treated myoblasts on flat BMGs and BMG-55s. Scale bars = 100 μm. (**j,k**) Transferrin/insulin-treated myoblasts cultured on BMGs for 72 hours in fusogenic media were fixed and stained for myosin heavy chain (MHC, green), which serves as a myoblast fusion marker^62^,^63^, and nuclei (blue). (**l**) Quantification of fusion index of myoblasts on flat BMGs and BMG-55s. Myoblasts on BMG-55s displayed significantly lower fusion index. Error bars represent standard error mean (SEM).*p ≤ 0.05 for significance, t-test, compared to corresponding values in flat BMG controls.

We observed that nanotopography-induced attenuation of cell fusion was correlated with reduced cytoskeletal remodeling. To probe nanotopography-induced changes in this process, we analyzed the differences in serum protein deposition on flat BMGs and BMG-55s (Supplementary Fig. S3). Atomic force microcopy (AFM) aided analysis of flat BMGs and nanopatterned BMGs prior to and following exposure to 10% serum-containing media suggested differences in protein deposition. Specifically, percent change in surface roughness following treatment was 45% for flat BMGs and 177% for BMG-55s (Supplementary Fig. S3). Furthermore, SEM analysis of macrophages revealed extensive fusion and formation of FBGCs. FBGCs formed extensions that interacted with individual macrophages, which is consistent with previous reports of macrophage fusion on other surfaces^52^,^53^ (Fig. 2a,c). In contrast, SEM of BMG-55s showed predominantly single cells, which demonstrated a distinct morphology with a raised cell center as shown in (Fig. 2b,d). We also observed the distinct change in cell spreading and elongation induced by nanotopography as early as 48 hours after culture with IL-4 using fluorescence microscopy (Fig. 2e,f). Cells on flat BMGs were found to be significantly larger and elongated as compared to cells on BMG-55s, suggesting that cytoskeletal remodeling is compromised in cells cultured on the latter (Fig. 2e–g).

**Figure 3:**
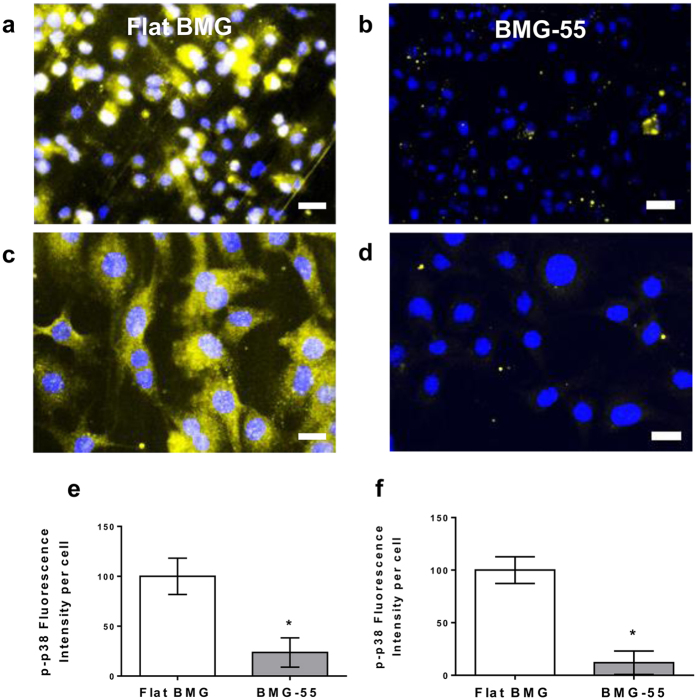
Compromised activation of p38 MAP Kinase induced by nanotopography. (**a,b**) Representative images of macrophages cultured on Flat BMGs and BMG-55s for one hour in IL-4 containing media and subsequently fixed and stained for p-p38 (yellow) and nuclei (blue). Scale bar = 21 μm. (**c,d**) Representative images of myoblasts cultured on Flat BMGs and BMG-55s for one hour with fusogenic media, fixed and stained for p-p38 (yellow) and nuclei (blue). Scale bar = 21 μm. (**e**) Quantification of total fluorescence intensity per cell of p-p38 in macrophages, expressed as a percentage of flat BMG value. Macrophages cultured on BMG-55s displayed significantly lower levels of p-p38. (**f**) Quantification of total fluorescence intensity per cell of p-p38 in myoblasts. Myoblasts cultured on BMG-55s displayed significantly lower levels of p-p38. Error bars represent standard error mean (SEM). *p ≤ 0.05 for significance, t-test, compared to corresponding values in flat BMG controls.

To further verify nanotopography-induced restriction of cytoskeletal remodeling, we investigated fusion of C2C12 myoblasts, which are fusogenic muscle progenitor cells^10^,^11^. BMG nanorod arrays induced a 50% reduction in myoblast fusion, which was similar to that observed for macrophages (Fig. 2h–l). In addition, SEM analysis revealed that cells on flat BMGs underwent extensive remodeling to form myotubes (Fig. 2h). In contrast, cells cultured on nanorod arrays showed minimal remodeling and were mostly individual cells (Fig. 2i). Based on our findings and published studies, we hypothesized that early activation of p38 MAP kinase might be involved in nanorod array-induced attenuation of cell fusion. Specifically, myogenesis was shown to require activation of p38 MAP Kinase and cells deficient in certain p38 isoforms displayed attenuated fusion capacity^54^,^55^,^56^. p38 MAP Kinase is also involved in the regulation of molecules such as P2×7, which regulate cytoskeletal remodeling in macrophage fusion^57^. Both macrophages (Fig. 3a,b,e) and myoblasts (Fig. 3c,d,f) cultured on BMG-55s with fusogenic media had lower levels of phospho-p38 as compared to flat BMGs. Collectively, our results indicate that nanotopography prevents the activation of p38 MAP Kinase, which limits cytoskeletal remodeling in fusogenic cells. Since early cytoskeletal remodeling is essential for cell fusion, we conclude that nanorod array-induced restriction of cell spreading and remodeling contributes to the altered fusion. The mechanism suggests that topography-induced modulation of cell fusion could be realized for other fusogenic cell types.

### *In vivo* evaluation of nanotopography-induced attenuation of cell fusion

**Figure 4:**
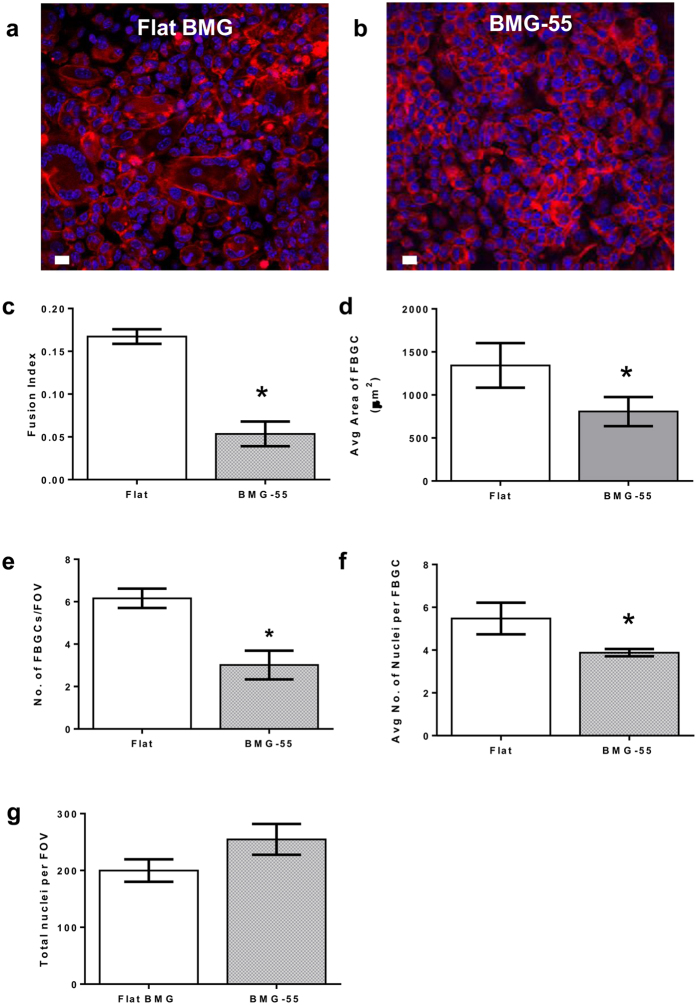
*In vivo* evaluation of FBR-associated cell-cell fusion on BMG nanorod arrays. Flat BMGs and BMG-55s were implanted IP in WT mice for 7 days. Explanted BMGs were stained and analyzed as described. **(a,b)** Explanted BMGs stained for F-actin (phalloidin) and nuclei (DAPI). Scale bar = 7 μm. **(c–g)** Quantification of fusion parameters. Fusion index was defined as the ratio of number of fused nuclei to total nuclei. BMG-55s resulted in attenuation of cell-cell fusion in *in vivo* settings. Fusion index, number of FBGCs, nuclei per FBGC and average area of FBGCs were significantly reduced on BMG-55s. Total number of nuclei per field of view was found to be larger on BMG-55s. (n = 6). Error bars represent standard error mean (SEM). *p ≤ 0.05 for significance, t-test, compared to corresponding values in flat BMG controls.

Furthermore, we evaluated nanotopography-mediated attenuation of FBR-associated cell fusion *in vivo* using an intraperitoneal (IP) FBGC formation model. Flat BMGs and BMG nanorod arrays were implanted in the intraperitoneal cavity of WT mice for 1 week. Explanted BMGs were stained and analyzed for cell fusion using confocal microscopy. Explanted BMG-55s had significantly lower number of FBGCs as compared to flat BMGs (Fig. 4a,b,e). Moreover, FBGCs on BMG-55s were smaller in size and had a smaller number of nuclei as compared to those on flat BMGs (Fig. 4a,b,d,f). Analysis revealed that 55 nm nanorod arrays attenuated FBR-associated cell fusion by over fifty percent (Fig. 4a–f).

## Discussion

Work presented here expands our fundamental understanding of the nanoscale biophysical regulation of cell-cell fusion. We have demonstrated the selective mechanosensitivity of cell fusion to nanotopographical features. Substrate nanotopography can override biochemical signals, to attenuate fusion. Biochemical induction of fusion in primary macrophages and myoblasts is mediated by extensive cytoskeletal remodeling, which enables cell-cell contact and fusion. Substrate nanotopography restricts the signaling necessary for early cytoskeletal remodeling in cells, thereby decreasing overall fusion in both cell types. Nanotopographical cues presented by ECM architecture are ubiquitous in nature and hence could be involved in regulation of cell fusion in other relevant cell types. Implications of nanotopography-mediated changes in cell fusion in physiology and disease progression remain to be evaluated.

Nevertheless, our current findings have implications in several areas of research because cell-cell fusion underlies cell-biomaterial interactions. Specifically, sensitivity of macrophage fusion to nanotopography can be exploited to attenuate biomaterial-induced FBR. As described before, progression of FBR is largely mediated by activated macrophages, which fuse on the surface of biomaterials to form FBGCs^9^. Macrophages and FBGCs release degradative factors such as ROS and pro-fibrotic signals^9^,^58^. Prolonged presence of these cells leads to infiltration by collagen-producing fibroblasts. The consequence of these events is the formation of a collagen-rich capsule, which isolates the biomaterial from its microenvironment. *In vitro* assays and *in viv*o experiments described here showed that 55 nm nanorod arrays attenuated macrophage fusion and formation of FBGCs. Incorporation of nanoscale architecture on the surface of biomaterials provides a unique means to limit FBR-associated cell-cell fusion, which could lead to improved biomaterial-tissue integration and performance. Hence, nanotopography-mediated attenuation of macrophage fusion can mitigate or delay biomaterial rejection and implant failure. We have previously shown that 55 nm nanorod arrays can induce decreased collagen production in fibroblasts^37^. Thus, 55 nm nanorod arrays can induce favorable cell responses in both macrophages and fibroblasts. Such nanorod arrays can be incorporated in biomaterial surface design to limit FBR towards tissue engineering constructs, biomedical devices and implants.

## Methods

### Primary Macrophage Isolation

Mouse macrophages were obtained as described previously^7^. Briefly, bone marrow was collected from mouse femurs. The mononuclear cell component was isolated using Lympholyte-M (Cedarlane Labs, Burlington, NC) as described previously^7^. Cells were plated in expansion medium (IMDM supplemented with 10% heat-inactivated fetal bovine serum (FBS), penicillin/streptomycin, 1.5 ng/ml M-CSF, and 100 ng/ml Flt3-ligand, fed on day 5 and collected on day 10 by scraping as described previously^7^.

### Fabrication of BMG nanorod arrays

Thermoplastic forming of BMGs was employed to form nanorod arrays with nanoporous alumina molds as described previously^35^,^37^,^59^,^60^. BMG nanorod arrays with diameters ranging from 55–200 nm were formed using commercially available (Whatman & Synkera) nanoporous alumina molds fabricated through a two-step anodization process. Additionally, three different BMG-55s were pressed with distinct applied force during the forming process. 35 kN, 15 kN and 5 kN were used to create BMG-55s with varying nanorod stiffness of 3 nN/nm, 14 nN/nm and 114 nN/nm respectively. Nanorod stiffness was calculated using Timoshenko’s beam theory. The beam theory describes the stiffness of each nanorod (*k*) as a function of nanorod length (*L*), nanorod radius (rrod), second moment of inertia of the nanorod (*I*), elastic modulus of the material (*E*) as well as the shear modulus of the material (G), and is given by Euler-Bernoulli’s beam theory as follows.


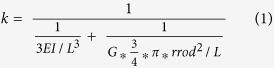


SEM was employed to inspect the samples for fidelity before use. Representative BMG samples were placed in endotoxin-free PBS for 1 week and the eluents were analyzed for endotoxin content with the Pyrogent Plus, 64 kit (Lonza, Allendale, NJ). All samples contained less than 0.06 EU/ml.

### *In Vitro* Macrophage Fusion & Analysis

Bone marrow-derived macrophages, were plated at a density of 260,000 cells/cm^2^ on ethanol-sterilized BMGs for 10 days in IMDM supplemented with 10% heat-inactivated FBS, penicillin/streptomycin, and 10 ng/ml IL-4. Media was replaced on Day 3, 5 and 7. At the end of 10 days, cells were fixed with 4% paraformaldehyde and stained with phalloidin and DAPI. Three/four independent samples were used for each type of BMG and at least ten images were analyzed for each sample. Image J was used for image analysis. For SEM samples, macrophages on BMGs were dehydrated using HMDS and serial ethanol washes followed by chrome-coating.

### *In Vitro* Myoblast Fusion

C2C12 myoblasts at a density of 20,000 cells/cm^2^ were cultured in media (DMEM with 10% serum, 1% Penicillin/streptomycin and 1% sodium pyruvate) on BMGs for 24 hours followed by change of media to include differentiation factors. DMEM with 1% heat-inactivated FBS, 5 μg/ml transferrin and 5 μg/ml insulin was used to induce cell-cell fusion as described previously^11^. Passages 1–3 after thawing out vials of C2C12 myoblast cell line were used.

At the end of 72 hours, cells were prepped either for SEM or for immunostaining with MHC, which is a myoblast fusion marker^11^. For staining, fixed cells were permeabilized using Triton and 5% normal goat serum/0.3% Triton was used for blocking. Cells were stained with an antibody against MHC (DHSB, MF20) and subsequently with an Alexa-488 secondary antibody for imaging. Three independent samples were used for each type of BMG and at least ten images were analyzed for each sample using Image J.

### Phospho-P38 in Macrophages And Myoblasts

Cells were seeded on BMGs and fixed using paraformaldehyde after 1 hour of culture with differentiation media. Primary Macrophages were seeded at a density of 260,000 cells/cm^2^ and C2C12s (passage 1–3) were cultured at a density of 20,000 cells/cm^2^. Fixed cells were stained for an antibody against phospho-p38 (Cell signaling, mAb #4631). A 488 nm fluorescent-tagged secondary antibody was used to visualize the protein and DAPI was used to label the nuclei. Three independent samples were used for each type of BMG & cell type and at least ten images were analyzed for each sample. Metamorph image analysis software was used to quantify fluorescence signal intensity normalized to number of nuclei. The fluorescence signal was converted to yellow to increase figure clarity.

### AFM analysis of BMGs

BMG samples were washed with ethanol for sterilization, rinsed with PBS and subsequently incubated in serum-containing media for three hours. BMGs were fixed with 4% paraformaldehyde and visualized using a Bruker AFM. For controls, flat BMGs and BMG-55s were analyzed after ethanol sterilization and rinsing with PBS.

### *In Vivo* Cell Fusion & Analysis

All procedures were performed in accordance with the regulations adopted by the National Institutes of Health and were approved by the Animal Care and Use Committee of Yale University. Intraperitoneal implantations were performed as described previously^7^. A total of 12 wild-type (WT) mice were used for the *in vivo* studies. Each mouse received a single BMG implanted in the peritoneum. Six independent samples were used for control flat BMGs and BMG-55s. BMGs remained in the peritoneum for 7 days prior to explantation. As described previously, intraperitoneal implants do not adhere to the omentum and can be easily retrieved and analyzed by staining^61^. Samples were then fixed and stained for image analysis. n = 6 mice were used for each BMG and at least ten images were analyzed for each sample.

## Additional Information

**How to cite this article**: Padmanabhan, J. *et al*. Regulation of cell-cell fusion by nanotopography. *Sci. Rep*. **6**, 33277; doi: 10.1038/srep33277 (2016).

## Supplementary Material

Supplementary Information

## References

[b1] Ogle B. M., Cascalho M. & Platt J. L. Biological implications of cell fusion. Nat Rev Mol Cell Biol 6, 567–575 (2005). 15957005 10.1038/nrm1678

[b2] Oren-Suissa M. & Podbilewicz B. Cell fusion during development. Trends Cell Biol 17, 537–546 (2007). 17981036 10.1016/j.tcb.2007.09.004

[b3] Pawelek J. M. & Chakraborty A. K. Fusion of tumour cells with bone marrow-derived cells: a unifying explanation for metastasis. Nat Rev Cancer 8, 377–386 (2008). 18385683 10.1038/nrc2371

[b4] Zhang L. . Zwitterionic hydrogels implanted in mice resist the foreign-body reaction. Nature biotechnology 31, 553–556 (2013). 10.1038/nbt.2580 23666011

[b5] Padmanabhan J. & Kyriakides T. R. Nanomaterials, inflammation, and tissue engineering. Wiley Interdiscip Rev Nanomed Nanobiotechnol 7, 355–370 (2015). 25421333 10.1002/wnan.1320 PMC4397128

[b6] MacLauchlan S. . Macrophage fusion, giant cell formation, and the foreign body response require matrix metalloproteinase 9. Journal of leukocyte biology 85, 617–626 (2009). 19141565 10.1189/jlb.1008588 PMC2656428

[b7] Moore L. B., Sawyer A. J., Charokopos A., Skokos E. A. & Kyriakides T. R. Loss of monocyte chemoattractant protein-1 alters macrophage polarization and reduces NF kappa B activation in the foreign body response. Acta Biomater 11, 37–47 (2015). 25242651 10.1016/j.actbio.2014.09.022 PMC4278755

[b8] Jay S. M., Skokos E. A., Zeng J., Knox K. & Kyriakides T. R. Macrophage fusion leading to foreign body giant cell formation persists under phagocytic stimulation by microspheres *in vitro* and *in vivo* in mouse models. Journal of biomedical materials research. Part A 93, 189–199 (2010). 19536825 10.1002/jbm.a.32513 PMC2826601

[b9] Anderson J. M., Rodriguez A. & Chang D. T. Foreign body reaction to biomaterials. Seminars in immunology 20, 86–100 (2008). 18162407 10.1016/j.smim.2007.11.004 PMC2327202

[b10] Shi H. . Improved regenerative myogenesis and muscular dystrophy in mice lacking Mkp5. J Clin Invest 123, 2064–2077 (2013). 23543058 10.1172/JCI64375 PMC3635719

[b11] Kontaridis M. I., Liu X., Zhang L. & Bennett A. M. SHP-2 complex formation with the SHP-2 substrate-1 during C2C12 myogenesis. J Cell Sci 114, 2187–2198 (2001). 11493654 10.1242/jcs.114.11.2187

[b12] Pajcini K. V., Pomerantz J. H., Alkan O., Doyonnas R. & Blau H. M. Myoblasts and macrophages share molecular components that contribute to cell-cell fusion. The Journal of cell biology 180, 1005–1019 (2008). 18332221 10.1083/jcb.200707191 PMC2265408

[b13] Jay S. M., Skokos E., Laiwalla F., Krady M. M. & Kyriakides T. R. Foreign body giant cell formation is preceded by lamellipodia formation and can be attenuated by inhibition of Rac1 activation. The American journal of pathology 171, 632–640 (2007). 17556592 10.2353/ajpath.2007.061213 PMC1934537

[b14] Gardner S., Gross S. M., David L. L., Klimek J. E. & Rotwein P. Separating myoblast differentiation from muscle cell fusion using IGF-I and the p38 MAP kinase inhibitor SB202190. Am J Physiol Cell Physiol, ajpcell 00184 02015 (2015). 10.1152/ajpcell.00184.2015 PMC4593770 26246429

[b15] Holt D. J. & Grainger D. W. Multinucleated giant cells from fibroblast cultures. Biomaterials 32, 3977–3987 (2011). 21397323 10.1016/j.biomaterials.2011.02.021 PMC3071287

[b16] Robinson J. M., Roos D. S., Davidson R. L. & Karnovsky M. J. Membrane alterations and other morphological features associated with polyethylene glycol-induced cell fusion. J Cell Sci 40, 63–75 (1979). 536393 10.1242/jcs.40.1.63

[b17] Lentz B. R. & Lee J. K. Poly(ethylene glycol) (PEG)-mediated fusion between pure lipid bilayers: a mechanism in common with viral fusion and secretory vesicle release? Mol Membr Biol 16, 279–296 (1999). 10766128 10.1080/096876899294508

[b18] Stevens M. M. & George J. H. Exploring and engineering the cell surface interface. Science 310, 1135–1138 (2005). 16293749 10.1126/science.1106587

[b19] Tan J. L. . Cells lying on a bed of microneedles: an approach to isolate mechanical force. Proc Natl Acad Sci USA 100, 1484–1489 (2003). 12552122 10.1073/pnas.0235407100 PMC149857

[b20] Tien J., Nelson C. M. & Chen C. S. Fabrication of aligned microstructures with a single elastomeric stamp. Proc Natl Acad Sci USA 99, 1758–1762 (2002). 11842197 10.1073/pnas.042493399 PMC122266

[b21] Dalby M. J., Pasqui D. & Affrossman S. Cell response to nano-islands produced by polymer demixing: a brief review. IEE Proc Nanobiotechnol 151, 53–61 (2004). 16475843 10.1049/ip-nbt:20040534

[b22] McMurray R. J. . Nanoscale surfaces for the long-term maintenance of mesenchymal stem cell phenotype and multipotency. Nat Mater 10, 637–644 (2011). 21765399 10.1038/nmat3058

[b23] Yim E. K., Darling E. M., Kulangara K., Guilak F. & Leong K. W. Nanotopography-induced changes in focal adhesions, cytoskeletal organization, and mechanical properties of human mesenchymal stem cells. Biomaterials 31, 1299–1306 (2010). 19879643 10.1016/j.biomaterials.2009.10.037 PMC2813896

[b24] Biggs M. J. . Interactions with nanoscale topography: adhesion quantification and signal transduction in cells of osteogenic and multipotent lineage. Journal of biomedical materials research. Part A 91, 195–208 (2009). 18814275 10.1002/jbm.a.32196

[b25] Ingham C. J., ter Maat J. & de Vos W. M. Where bio meets nano: the many uses for nanoporous aluminum oxide in biotechnology. Biotechnol Adv 30, 1089–1099 (2012). 21856400 10.1016/j.biotechadv.2011.08.005

[b26] Dalby M. J. . Increasing fibroblast response to materials using nanotopography: morphological and genetic measurements of cell response to 13-nm-high polymer demixed islands. Exp Cell Res 276, 1–9 (2002). 11978003 10.1006/excr.2002.5498

[b27] Jeon H., Simon C. G. Jr. & Kim G. A mini-review: Cell response to microscale, nanoscale, and hierarchical patterning of surface structure. Journal of biomedical materials research. Part B, Applied biomaterials 102, 1580–1594 (2014). 24678035 10.1002/jbm.b.33158

[b28] Yang H. S. . Electroconductive Nanopatterned Substrates for Enhanced Myogenic Differentiation and Maturation. Adv Healthc Mater 5, 137–145 (2016). 25988569 10.1002/adhm.201500003 PMC5003176

[b29] Hosseini V. . Engineered contractile skeletal muscle tissue on a microgrooved methacrylated gelatin substrate. Tissue engineering. Part A 18, 2453–2465 (2012). 22963391 10.1089/ten.tea.2012.0181 PMC3501120

[b30] Chen S. . Characterization of topographical effects on macrophage behavior in a foreign body response model. Biomaterials 31, 3479–3491 (2010). 20138663 10.1016/j.biomaterials.2010.01.074 PMC2837101

[b31] Lamers E. . *In vitro* and *in vivo* evaluation of the inflammatory response to nanoscale grooved substrates. Nanomedicine 8, 308–317 (2012). 21704595 10.1016/j.nano.2011.06.013

[b32] Neacsu P. . Reduced inflammatory activity of RAW 264.7 macrophages on titania nanotube modified Ti surface. Int J Biochem Cell Biol 55, 187–195 (2014). 25220343 10.1016/j.biocel.2014.09.006

[b33] Majd H. . Novel micropatterns mechanically control fibrotic reactions at the surface of silicone implants. Biomaterials 54, 136–147 (2015). 25907047 10.1016/j.biomaterials.2015.03.027

[b34] Li H. F. & Zheng Y. F. Recent advances in bulk metallic glasses for biomedical applications. Acta Biomater 36, 1–20 (2016). 27045349 10.1016/j.actbio.2016.03.047

[b35] Kumar G., Tang H. X. & Schroers J. Nanomoulding with amorphous metals. Nature 457, 868–872 (2009). 19212407 10.1038/nature07718

[b36] Schroers J., Kumar G., Hodges T. M., Chan S. & Kyriakides T. R. Bulk metallic glasses for biomedical applications. Jom-Us 61, 21–29 (2009).

[b37] Padmanabhan J. . Engineering cellular response using nanopatterned bulk metallic glass. ACS nano 8, 4366–4375 (2014). 24724817 10.1021/nn501874q PMC4046793

[b38] Huang L., Zhang T., Liaw P. K. & He W. Macrophage responses to a Zr-based bulk metallic glass. Journal of biomedical materials research. Part A 102, 3369–3378 (2014). 24166768 10.1002/jbm.a.35009

[b39] Huang L. . Ni-free Zr-Cu-Al-Nb-Pd bulk metallic glasses with different Zr/Cu ratios for biomedical applications. J Biomed Mater Res B Appl Biomater 100, 1472–1482 (2012). 22689253 10.1002/jbm.b.32715

[b40] Schroers J. . Thermoplastic blow molding of metals. Mater Today 14, 14–19 (2011).

[b41] Ding S. . Combinatorial development of Metallic Glasses. Nature Materials 13, 494 (2014). 24728462 10.1038/nmat3939

[b42] Discher D. E., Janmey P. & Wang Y. L. Tissue cells feel and respond to the stiffness of their substrate. Science 310, 1139–1143 (2005). 16293750 10.1126/science.1116995

[b43] Kam K. R. . The effect of nanotopography on modulating protein adsorption and the fibrotic response. Tissue engineering. Part A 20, 130–138 (2014). 23914986 10.1089/ten.tea.2012.0772 PMC3875207

[b44] Branco da Cunha C. . Influence of the stiffness of three-dimensional alginate/collagen-I interpenetrating networks on fibroblast biology. Biomaterials 35, 8927–8936 (2014). 25047628 10.1016/j.biomaterials.2014.06.047

[b45] Pixley F. J. Macrophage Migration and Its Regulation by CSF-1. Int J Cell Biol 2012, 501962 (2012). 22505929 10.1155/2012/501962 PMC3296313

[b46] Evans J. G., Correia I., Krasavina O., Watson N. & Matsudaira P. Macrophage podosomes assemble at the leading lamella by growth and fragmentation. J Cell Biol 161, 697–705 (2003). 12756237 10.1083/jcb.200212037 PMC2199349

[b47] Bashur C. A., Shaffer R. D., Dahlgren L. A., Guelcher S. A. & Goldstein A. S. Effect of fiber diameter and alignment of electrospun polyurethane meshes on mesenchymal progenitor cells. Tissue engineering. Part A 15, 2435–2445 (2009). 19292650 10.1089/ten.tea.2008.0295

[b48] Xie J. . Radially aligned, electrospun nanofibers as dural substitutes for wound closure and tissue regeneration applications. ACS nano 4, 5027–5036 (2010). 20695478 10.1021/nn101554u PMC2947607

[b49] Schinwald A., Chernova T. & Donaldson K. Use of silver nanowires to determine thresholds for fibre length-dependent pulmonary inflammation and inhibition of macrophage migration *in vitro*. Part Fibre Toxicol 9, 47 (2012). 23199075 10.1186/1743-8977-9-47 PMC3546062

[b50] Cao H., McHugh K., Chew S. Y. & Anderson J. M. The topographical effect of electrospun nanofibrous scaffolds on the *in vivo* and *in vitro* foreign body reaction. Journal of biomedical materials research. Part A 93, 1151–1159 (2010). 19768795 10.1002/jbm.a.32609 PMC9069486

[b51] Albanese A., Tang P. S. & Chan W. C. The effect of nanoparticle size, shape, and surface chemistry on biological systems. Annu Rev Biomed Eng 14, 1–16 (2012). 22524388 10.1146/annurev-bioeng-071811-150124

[b52] McNally A. K. & Anderson J. M. Beta1 and beta2 integrins mediate adhesion during macrophage fusion and multinucleated foreign body giant cell formation. The American journal of pathology 160, 621–630 (2002). 11839583 10.1016/s0002-9440(10)64882-1 PMC1850662

[b53] McNally A. K., Macewan S. R. & Anderson J. M. Foreign body-type multinucleated giant cell formation requires protein kinase C beta, delta, and zeta. Exp Mol Pathol 84, 37–45 (2008). 18067888 10.1016/j.yexmp.2007.10.005 PMC2275167

[b54] Perdiguero E. . Genetic analysis of p38 MAP kinases in myogenesis: fundamental role of p38alpha in abrogating myoblast proliferation. EMBO J 26, 1245–1256 (2007). 17304211 10.1038/sj.emboj.7601587 PMC1817635

[b55] Wu Z. . p38 and extracellular signal-regulated kinases regulate the myogenic program at multiple steps. Molecular and cellular biology 20, 3951–3964 (2000). 10805738 10.1128/mcb.20.11.3951-3964.2000 PMC85749

[b56] Bhatnagar S., Kumar A., Makonchuk D. Y., Li H. & Kumar A. Transforming growth factor-beta-activated kinase 1 is an essential regulator of myogenic differentiation. The Journal of biological chemistry 285, 6401–6411 (2010). 20037161 10.1074/jbc.M109.064063 PMC2825435

[b57] Pfeiffer Z. A. . The nucleotide receptor P2X7 mediates actin reorganization and membrane blebbing in RAW 264.7 macrophages via p38 MAP kinase and Rho. Journal of leukocyte biology 75, 1173–1182 (2004). 15075366 10.1189/jlb.1203648

[b58] ten Harkel B. . The Foreign Body Giant Cell Cannot Resorb Bone, But Dissolves Hydroxyapatite Like Osteoclasts. PloS one 10, e0139564 (2015). 26426806 10.1371/journal.pone.0139564 PMC4591016

[b59] Carmo M. . Bulk metallic glass nanowire architecture for electrochemical applications. ACS nano 5, 2979–2983 (2011). 21370891 10.1021/nn200033c

[b60] Mukherjee S. . Tunable Hierarchical Metallic-Glass Nanostructures. Adv Funct Mater 23, 2708–2713 (2013).

[b61] Moore L. B., Sawyer A. J., Saucier-Sawyer J., Saltzman W. M. & Kyriakides T. R. Nanoparticle delivery of miR-223 to attenuate macrophage fusion. Biomaterials 89, 127–135 (2016). 26967647 10.1016/j.biomaterials.2016.02.036 PMC4924476

[b62] Fornaro M. . SHP-2 activates signaling of the nuclear factor of activated T cells to promote skeletal muscle growth. The Journal of cell biology 175, 87–97 (2006). 17015617 10.1083/jcb.200602029 PMC2064501

[b63] Ostrovidov S. . Three-dimensional co-culture of C2C12/PC12 cells improves skeletal muscle tissue formation and function. J Tissue Eng Regen Med (2014). 10.1002/term.1956 25393357

