# Supplementary material for: Regulation of cell-cell fusion by nanotopography
Source: Sci Rep. 2016 Sep 12;6:33277. doi: 10.1038/srep33277 (PMC5018837; doi:10.1038/srep33277)
Supplement: Supplementary Information [file srep33277-s1.pdf]

Supplementary Information

Regulation of cell-cell fusion by nanotopography

Authors

Jagannath Padmanabhan[1,2], Michael J. Augelli[1], Bettina Cheung[1,2], Emily R. Kinser[1,3,4] , Barnett Cleary[1], Priyanka Kumar[1], Renhao Wang[1], Andrew J. Sawyer[5], Rui Li[1], Udo D. Schwarz[1,3], Jan Schroers[1,3] & Themis R. Kyriakides*[1,2,5]

Affiliations

1Center for Research on Interface Structures and Phenomena, Yale University, New Haven, CT 06520, USA

2Dept. of Biomedical Engineering, Yale University, New Haven, CT 06520, USA

3Dept. of Mechanical Engineering and Materials Science, Yale University, New Haven, CT 06520, USA

4IBM Semiconductor Research & Development Center/IBM Corporate, Patent Engineer

5Department of Pathology, Yale University, New Haven, CT 06520, USA

*Corresponding Author:

Themis R. Kyriakides, Ph.D. Campus Address:

Associate Professor of Pathology and Biomedical Engineering

10 Amistad, Rm. 301C , Yale School of Medicine

New Haven, CT 06520

Email: themis.kyriakides@yale.edu

Keywords*:* cell-cell fusion, foreign body response, nanotopography, stiffness, bulk metallic glass.

a

BMG Nanorod Array (nominal rod diameter in nm)	Nanorod Diameter (nm)	Nanorod Height (nm)	Nanorod Aspect Ratio (length:diameter)	Nanorod Stiffness (nN/nm)
Flat BMG	NA	NA	NA	NA
BMG-55	64 ± 10	440 ± 106	6.88	2.75
BMG-100	113 ± 14	1103 ± 146	9.76	1.69
BMG-150	181 ± 18	1134 ± 210	6.27	10.27
BMG-200	247 ± 27	1755 ± 214	7.11	9.61

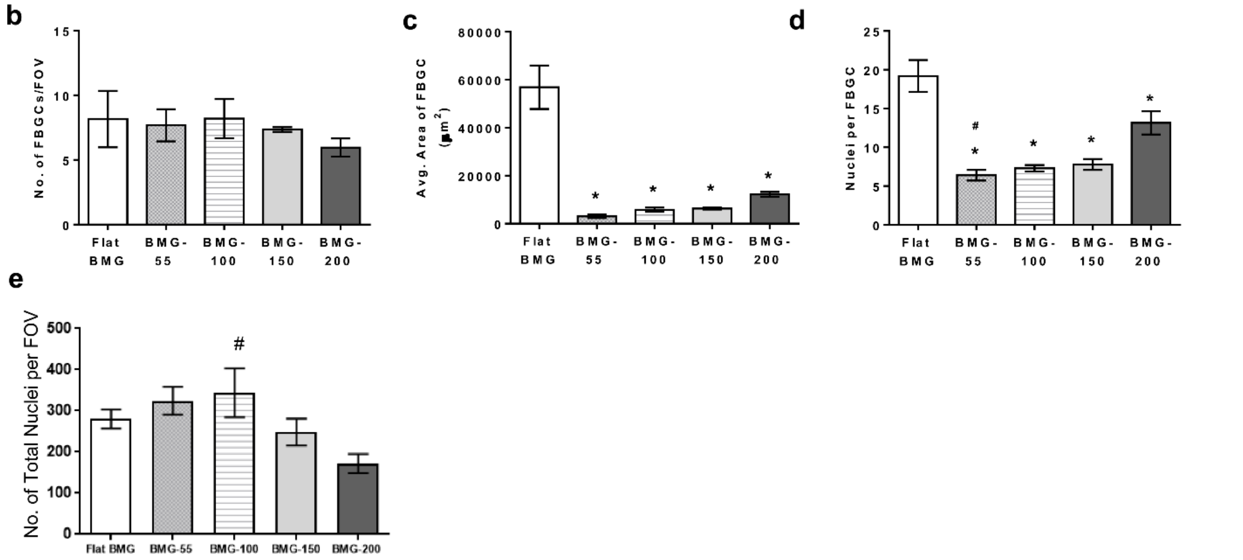

Fig.S1: Effect of nanotopography on cell fusion (**a**) BMG nanorod arrays with varied topography were generated using nanoporous alumina molds as templates. Table showing average nanorod diameter, length and stiffness for each BMG nanorod array. Primary bone-marrow derived murine macrophages were cultured on the nanorod arrays and cell fusion was biochemically induced. Image analysis enabled quantification of **(b)** Number of FBGCs, **(c)** FBGC size and **(d)** nuclei per FBGC. **(e)** Total nuclei per field of view. Error bars represent standard error mean (SEM) * represents significant differences as compared to flat BMGs. # represents significant differences as compared to BMG-200. (ANOVA with Tukey's post-hoc analysis, n ≥ 3, p ≤ 0.05 for significance.)

a

BMG Nanorod Array	Max. Force applied during TPF (kN)	Nanorod Height (nm)	Nanorod Aspect Ratio (length:diameter)	Nanorod Stiffness (nN/nm)
BMG-55 (AR = 7)	35	440 ± 106	6.88	2.75
BMG-55 (AR = 4)	15	257 ± 50	4.02	13.79
BMG-55 (AR = 2)	5	127 ± 28	1.98	114.34

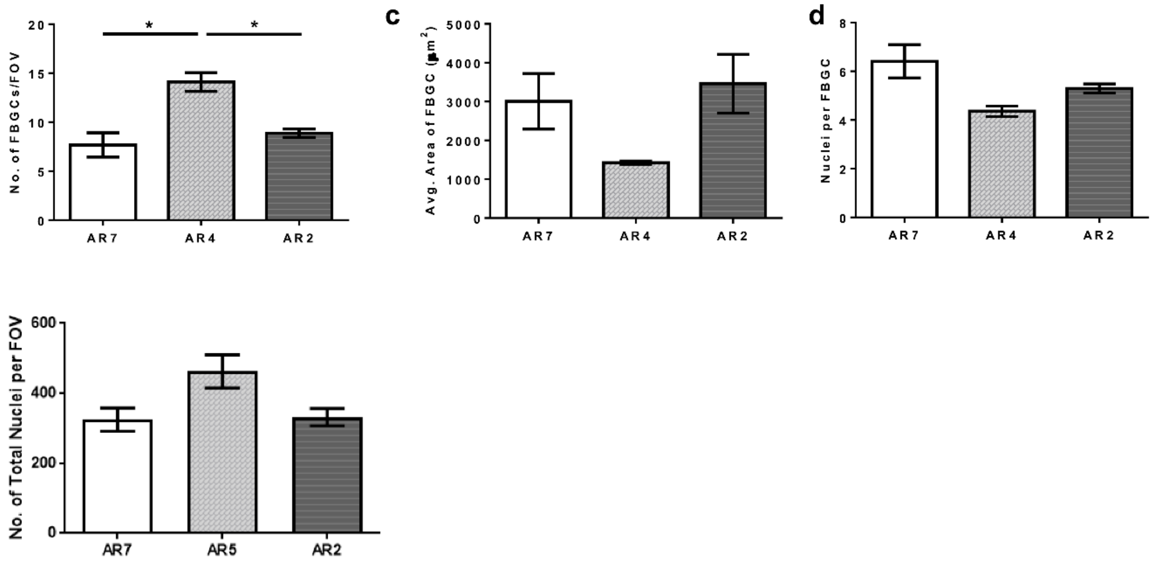

<u>Fig. S2: Effect of nanorod stiffness on cell-cell fusion</u> (**a**) BMG nanorod arrays with similar topography and varied stiffness were generated using different forming pressures. Nanorod diameters for all BMG-55s were 64±10 nm. Nanorod aspect ratio varied from 7-2. Table showing average nanorod length and stiffness for each BMG nanorod array generated. Primary bone-marrow derived murine macrophages were cultured on the nanorod arrays and cell fusion was biochemically induced. . Image analysis enabled quantification of **(b)** Number of FBGCs, **(c)** FBGC size and **(d)** nuclei per FBGC. **(e)** Total nuclei per field of view. Error bars represent standard error mean (SEM) * represents significant differences. (ANOVA with Tukey's post-hoc analysis, n ≥ 3, p ≤ 0.05 for significance.)

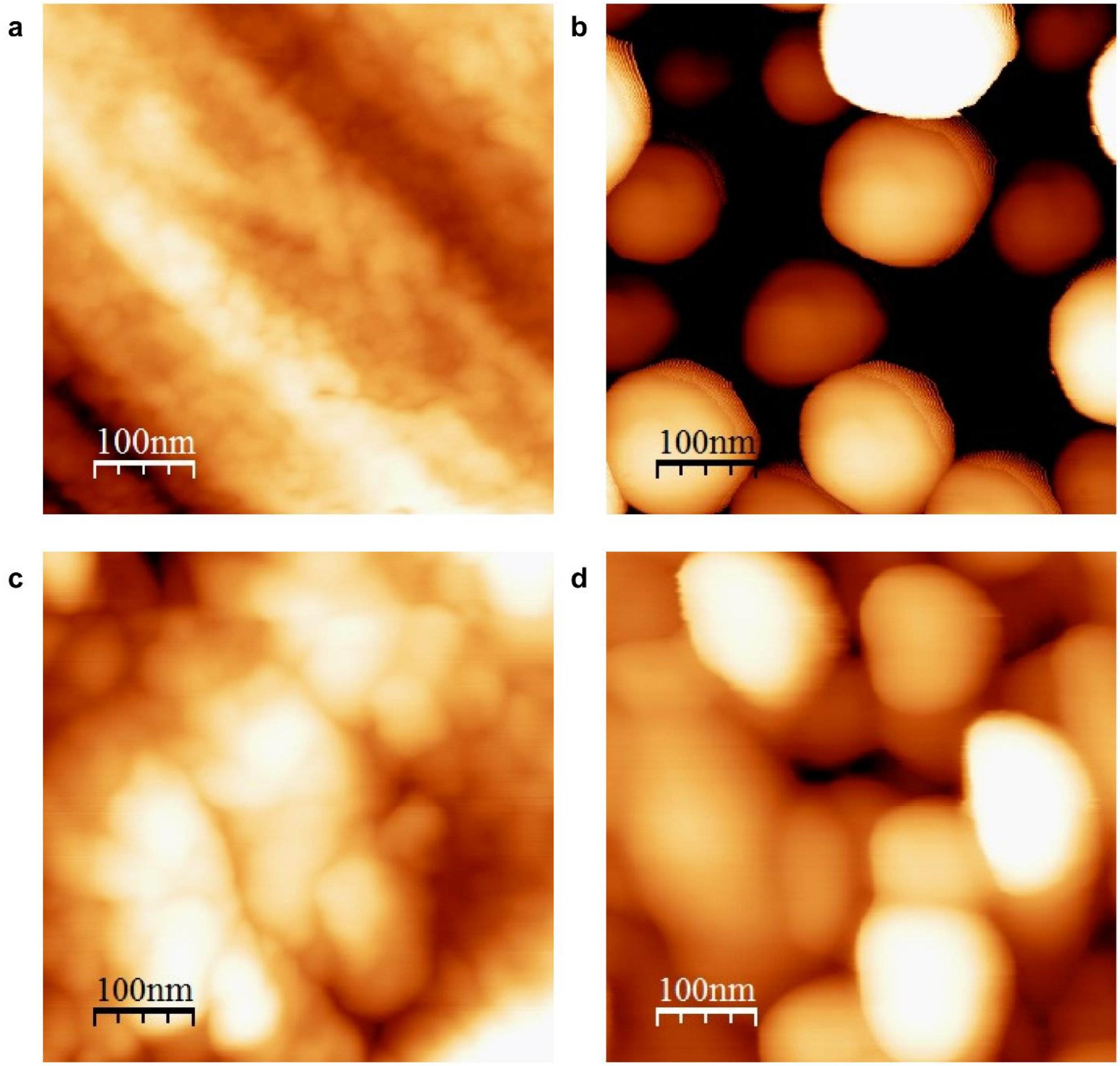

<u>Fig.S3: AFM imaging of BMGs</u> Flat BMGs (**a , c**) and BMG-55s (**b , d**) are shown. Images in **c** and **d** show BMGs incubated with serum-containing media prior to AFM whereas **a** and **b** were untreated. Scale Bar = 100 nm (**a-d**). Surface Roughness or RMS values for untreated and treated flat BMGs and the top surface of BMG-55s were found to be within 1-12 nm, much smaller than the nanopattern feature sizes studied here. Percent change in RMS induced by serum protein deposition was 45% for flat BMGs and 177% for BMG-55s.
